# Supplementary material for: Comparative Analysis of SLA-1, SLA-2, and DQB1 Genetic Diversity in Locally-Adapted Kenyan Pigs and Their Wild Relatives, Warthogs
Source: Vet Sci. 2021 Sep 2;8(9):180. doi: 10.3390/vetsci8090180 (PMC8473215; doi:10.3390/vetsci8090180)
Supplement: Supplementary file 1 [file vetsci-08-00180-s001.zip › Supplementary Data/Table S1_Primer details.pdf]

Table S1: The locus-specific primers for reverse transcription-polymerase chain reaction (RT-PCR) based method for DNA sequence-based typing (SBT) of the three SLA genes.

Locus	Primer name	Primer sequence (5'-3')	Annealing Temperature (T_a) °C	Amplicon size (bp)	Target Domain (s)	Reference
SLA-1	T7-SLA-alpha-F	TAATACGACTCACTATAGGGACCGCCGTGTCCCGGC(C/T)CGAC	60	280	Exon 2 (alpha 1)	This study.
	SP6-SLA-Alpha-R	ATTTAGGTGACACTATAGCCTCGCTCTGGTTGTAGTAGC				
SLA-2	*SLA-2*_F	CCACAGAATCTCCGCAGATTCC	65	1232	Exons 1-8<br>(all *SLA-2* domains)	(Gao *et al.*, 2014)
	*SLA-2*_R	CCGACACAGACACATTCAAATGCT				
DQB1	*DQB1* -F	GGGATAGGAGAACCACTGAG	66	917	Exons 1-5<br>(all *DQB1* domains)	(Gao *et al.*, 2014)
	DQB1 -R	AATTCCAGGCAAGAACAGAC				
