# Supplementary material for: Comparative Analysis of SLA-1, SLA-2, and DQB1 Genetic Diversity in Locally-Adapted Kenyan Pigs and Their Wild Relatives, Warthogs
Source: Vet Sci. 2021 Sep 2;8(9):180. doi: 10.3390/vetsci8090180 (PMC8473215; doi:10.3390/vetsci8090180)
Supplement: Supplementary file 1 [file vetsci-08-00180-s001.zip › Supplementary Data/Table S3_Peptide positions predictions for SLA-2 and DQB1.pdf]

Supplementary Table S3A: The polypeptide predictions for the *SLA-2* sequences.

region of interest	Domain name	refseq accession	original feature annotation in the refseq (nucl)		columns in largest_compact_alignment		num. codons
			begin	end	begin	end	
**largest alignment**		AJ251829.1			1	1056	352
**exon 1**	Peptide leader	AJ251829.1	144585	144648	1	31	10
**exon 2 (PBR)**	α1	AJ251829.1	144963	145232	32	304	91
**exon 3 (PBR)**	α2	AJ251829.1	145460	145735	305	580	92
**exon 4**	α3	AJ251829.1	146344	146619	581	856	92
**exon 5**	Transmembrane domain	AJ251829.1	146739	146849	857	967	37
**exon 6**	Cytoplasmic tail	AJ251829.1	147294	147326	968	1000	11
**exon 7**	Cytoplasmic tail	AJ251829.1	147454	147504	1001	1051	17
**exon 8**	3’ UTR	AJ251829.1	147665	147669	1052	1056	2

Supplementary Table S3B: The SLA polypeptide predictions for the *DQB1* sequences.

Region of interest	Domain name	Refseq accession	Original feature annotation in the RefSeq (Nucl)		Region spanned by our data with reference to the RefSeq (Nucl)		columns in largest_compact_alignment		No of codons
			begin	end	begin	end	begin	end	
**Largest alignment**		NM_001113694.1			82	840	1	759	253
**Exon 1**	**Peptide leader**	NM_001113694.1	1	160	82	160	1	79	26
**Exon 2 (PBR)**	**β1**	NM_001113694.1	161	430	161	430	80	349	90
**Exon 3**	**β2**	NM_001113694.1	431	712	431	712	350	631	94
**Exon 4**	**Transmembrane domain**	NM_001113694.1	713	823	713	823	632	742	37
**Exon 5**	**Cytoplasmic tail**	NM_001113694.1	824	1194	824	840	743	759	5.67
