# Supplementary figures and images for: Comparative Analysis of SLA-1, SLA-2, and DQB1 Genetic Diversity in Locally-Adapted Kenyan Pigs and Their Wild Relatives, Warthogs
Source: Vet Sci. 2021 Sep 2;8(9):180. doi: 10.3390/vetsci8090180 (PMC8473215; doi:10.3390/vetsci8090180)

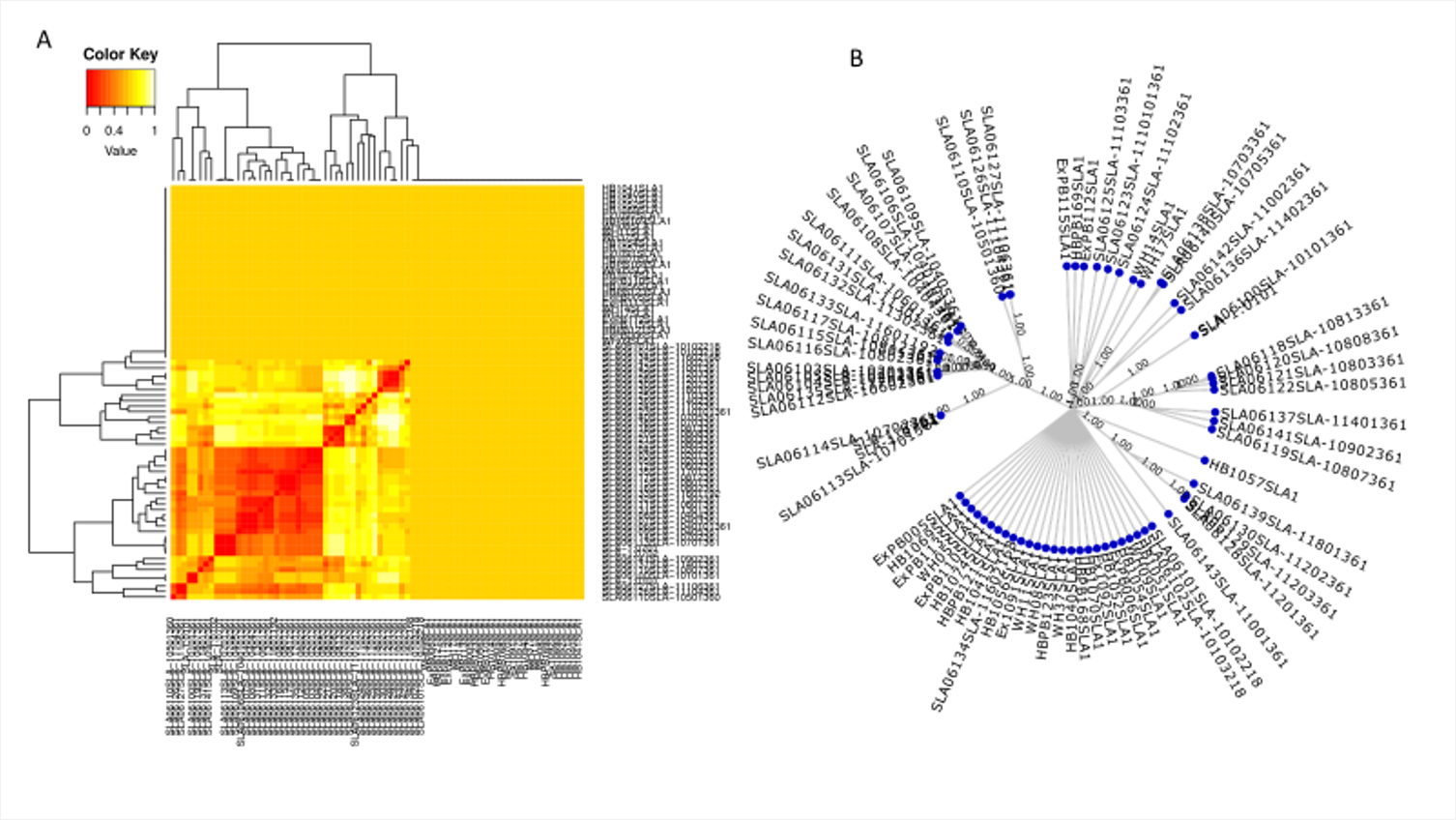

Supplement: Supplementary file 1 [file vetsci-08-00180-s001.zip › Supplementary Data/Figure S1_SLA1 MHCcluster heatmap and tree.png]
